# Conserved skeletal muscle transcriptomic responses to pacing strategies in Thoroughbred horses

**DOI:** 10.1242/bio.062724

**Published:** 2026-07-17

**Authors:** Kenya Takahashi, Kazutaka Mukai, Takanaga Shirai, Yusaku Ebisuda, Fumi Sugiyama, Toshinobu Yoshida, Hideo Hatta, Yu Kitaoka

**Affiliations:** ^1^ Department of Sports Sciences, The University of Tokyo, Tokyo 153-8902, Japan; ^2^ Sports Science Division, Equine Research Institute, Japan Racing Association, Tochigi 329-0412, Japan; ^3^ Department of Human Sciences, Kanagawa University, Kanagawa 221-8686, Japan

**Keywords:** High-intensity exercise, RNA-sequencing, Pacing strategy, Skeletal muscle, Thoroughbred

## Abstract

This study investigated whether different pacing patterns during high-intensity exercise elicit distinct transcriptional responses in equine skeletal muscle. Eight Thoroughbred horses completed two treadmill exercise sessions in a randomized crossover design. In the positive-pacing condition, horses exercised at 110% maximal O_2_ uptake (V̇O_2_max) for 1 min followed by 90% V̇O_2_max for 1 min, whereas in the negative-pacing condition, the order was reversed. At 4 h after exercise, the positive-pacing protocol resulted in upregulation of 1989 genes and downregulation of 840 genes, whereas the negative-pacing protocol resulted in upregulation of 1710 genes and downregulation of 593 genes (false discovery rate <0.05; fold change ≥1.5). Despite these differences, most exercise-responsive genes and pathways related to hypoxia signaling, extracellular matrix remodeling, and metabolic regulation were shared between protocols. A direct comparison of gene expression between the two protocols identified four genes with higher expression after positive pacing, including *RP1*, *MORN5*, and two unannotated equine transcripts (ENSECAG00000060378 and ENSECAG00000057614), whereas five genes (*OLFML2B*, *POSTN*, *ANGPTL1*, *MAP1A*, and *ZNF554*) showed higher expression after negative pacing. These findings indicate that the skeletal muscle transcriptomic response to workload-matched high-intensity exercise is largely conserved between pacing strategies in Thoroughbred horses, with only limited pacing-dependent differences.

## Teaser

**Summary:** This study shows that different pacing patterns during intense exercise produce broadly similar muscle gene responses in racehorses, with only limited differences.

## INTRODUCTION

Skeletal muscle adapts to exercise training through coordinated transcriptional programs that regulate metabolism, structure, and cellular remodeling (Egan and Zierath, 2013). Acute bouts of high-intensity exercise are potent stimuli for these transcriptional responses, initiating molecular processes that underlie long-term training adaptations. Although exercise intensity is known to be a key determinant of cellular events governing skeletal muscle adaptations (Egan et al., 2010), exercise intensity is not necessarily constant in competitive training. Indeed, training sessions often incorporate deliberate variations in intensity, such as a faster initial phase or progressive acceleration toward the end of an exercise bout. Positive (fast-start) and negative (slow-start) pacing strategies represent different patterns of intensity distribution within an exercise bout, and fast-start pacing has been shown to alter oxygen uptake kinetics and exercise tolerance even when total work is matched (Jones et al., 2008). However, whether pacing-dependent physiological differences during acute exercise are accompanied by distinct transcriptional responses in skeletal muscle remains unclear.

Thoroughbred horses provide a well-established large-animal model in which high-intensity exercise can be imposed under highly controlled treadmill conditions, with exercise intensity prescribed relative to each individual's maximal oxygen uptake (V̇O_2_max) (Kitaoka et al., 2011; Poole and Erickson, 2011). Moreover, the crossover design used in the present study enabled within-horse comparison of the two pacing strategies, thereby reducing the influence of inter-individual variability. A 2-min bout of high-intensity exercise was chosen to reflect the timescale of Thoroughbred racing, which was shown to be sufficient to induce substantial glycogen breakdown and lactate accumulation in equine skeletal muscle (Harris et al., 1987; Kitaoka et al., 2014). We previously showed that positive and negative pacing induced distinct skeletal muscle metabolic responses despite identical total exercise duration and workload, with positive pacing associated with greater glycogen depletion (Takahashi et al., 2024). Based on these previous metabolomic findings, we hypothesized that positive pacing would elicit a more pronounced post-exercise transcriptomic response in equine skeletal muscle. The 4-h post-exercise time point was selected because previous equine studies have shown robust gene expression changes at this stage (Bryan et al., 2017; McGivney et al., 2009), and human studies also identify 3–4 h after exercise as a key window for detecting mRNA responses (Kuang et al., 2022; Popov et al., 2019). Therefore, the present study examined skeletal muscle transcriptomic responses 4 h after a 2-min bout of high-intensity treadmill exercise performed with positive- or negative-pacing strategies.

## RESULTS

### Multivariate analysis

**Fig. 1.**
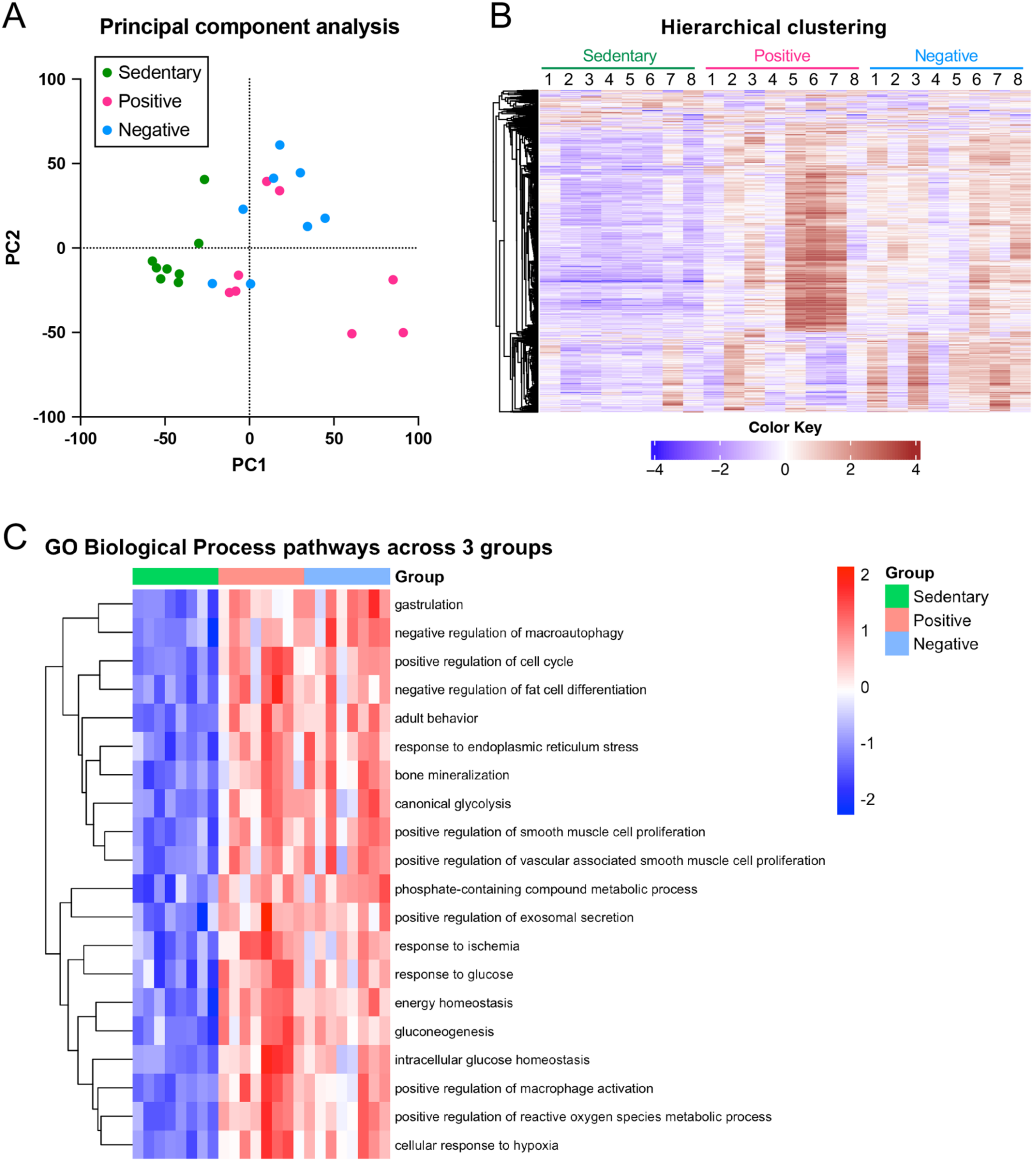
**Global transcriptomic responses of equine skeletal muscle to high-intensity exercise with different intensity distributions.** (A) PCA of normalized gene expression profiles showing global transcriptomic differences among sedentary (green), positive pacing (pink), and negative pacing (blue) conditions. Each point represents an individual sample. (B) Hierarchical clustering heatmap of the top 1000 most variable genes across all samples. Gene expression values were centered and scaled by gene. Samples are grouped and annotated according to experimental condition, with individual animal identifiers shown above the heatmap. (C) Heatmap of the top 20 GO BP pathways showing pathway-level expression patterns across the three conditions. Pathway activity scores were calculated using single-sample gene set enrichment analysis (ssGSEA), centered and scaled across samples for each pathway, and visualized by hierarchical clustering of pathways. Colors indicate relative expression or pathway activity levels, with blue and red representing lower and higher values, respectively.

Principal component analysis (PCA) was performed to visualize global differences in gene expression profiles among the experimental conditions (Fig. 1A). Samples from the sedentary condition clustered distinctly from those obtained after exercise along PC1, indicating a pronounced exercise-induced shift in the skeletal muscle transcriptome. By comparison, samples from the positive- and negative-pacing conditions exhibited only partial separation with overlap, suggesting that pacing-dependent differences were smaller than the overall exercise effect.

To further examine similarities and differences across samples, hierarchical clustering was performed using the top 1000 most variable genes in an unsupervised manner (Fig. 1B). The resulting heatmap showed clear segregation between sedentary and exercised samples, whereas samples from the two exercise conditions clustered close to one another. These multivariate analyses indicate that high-intensity exercise was the primary driver of the global transcriptomic response.

To gain insight into the biological processes associated with these condition-dependent expression patterns, we performed Gene Ontology biological process (GO BP) gene set enrichment analysis (Table S1). The heatmap of the top 20 pathways showed robust enrichment in both exercise conditions compared with the sedentary reference condition, including processes related to glucose metabolism, energy homeostasis, cellular stress responses, hypoxia, reactive oxygen species metabolism, and macrophage activation (Fig. 1C).

### Differential gene expression induced by high-intensity exercise

**Fig. 2.**
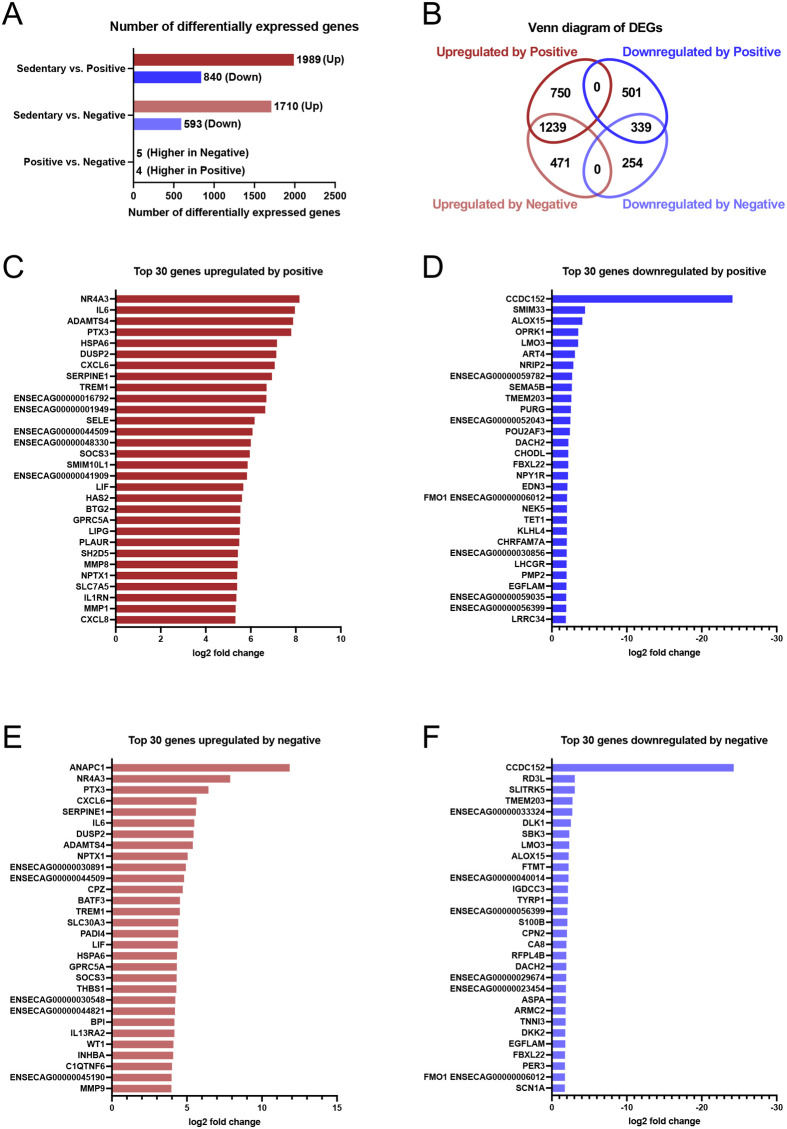
**Differential gene expression profiles induced by different exercise intensity distributions.** (A) Number of DEGs identified in pairwise comparisons between sedentary and exercised conditions, and between positive and pacing patterns. Bars indicate the number of genes upregulated (red) or downregulated (blue) in each comparison. (B) Venn diagram illustrating the overlap of DEGs upregulated or downregulated by positive and negative pacing relative to the sedentary condition. (C,D) Top 30 genes upregulated (C) and downregulated (D) by the positive pacing relative to the sedentary condition, ranked by log_2_ fold change. (E,F) Top 30 genes upregulated (E) and downregulated (F) by the negative pacing relative to the sedentary condition, ranked by log_2_ fold change. All differential expression analyses were performed using RNA-seq data with appropriate normalization and multiple testing correction. Log_2_ fold change values are shown for each gene.

Differential expression analysis revealed robust transcriptional responses to high-intensity exercise compared with the sedentary condition (Fig. 2A). Compared with the sedentary condition, the positive pacing resulted in 1989 upregulated and 840 downregulated genes, whereas the negative pacing resulted in 1710 upregulated and 593 downregulated genes. In contrast, direct comparison between the two exercise protocols identified only a small number of differentially expressed genes, with four genes expressed at higher levels in the positive-pacing condition and five genes expressed at higher levels in the negative-pacing condition.

Venn diagram analysis further illustrated the extent of overlap in exercise-responsive genes between the two pacing conditions (Fig. 2B). Genes appearing in the non-overlapping sections of the Venn diagram represented genes that were significantly differentially expressed relative to the pre-exercise reference condition in one exercise condition but did not meet the differential expression criteria in the other condition. Numerous genes that were upregulated (*n*=1239) or downregulated (*n*=339) after exercise were common to both pacing conditions, indicating that most exercise-responsive genes were shared.

To further characterize genes contributing most strongly to exercise-induced transcriptional changes, the top 30 upregulated and downregulated genes in each exercise condition were examined (Fig. 2C–F). Of the top 30 upregulated genes, 14 were shared between positive and negative pacing, including *NR4A3*, *IL6*, *ADAMTS4*, *PTX3*, *HSPA6*, *DUSP2*, *CXCL6*, *SERPINE1*, *TREM1*, *SOCS3*, *LIF*, *GPRC5A*, and *NPTX1*. Of the top 30 downregulated genes, nine were shared between pacing conditions, including *CCDC152*, *ALOX15*, *LMO3*, *TMEM203*, *DACH2*, *FBXL22*, *FMO1*, *EGFLAM*, and *ENSECAG00000056399*.

### Limited pacing-dependent differences in gene expression

**Fig. 3.**
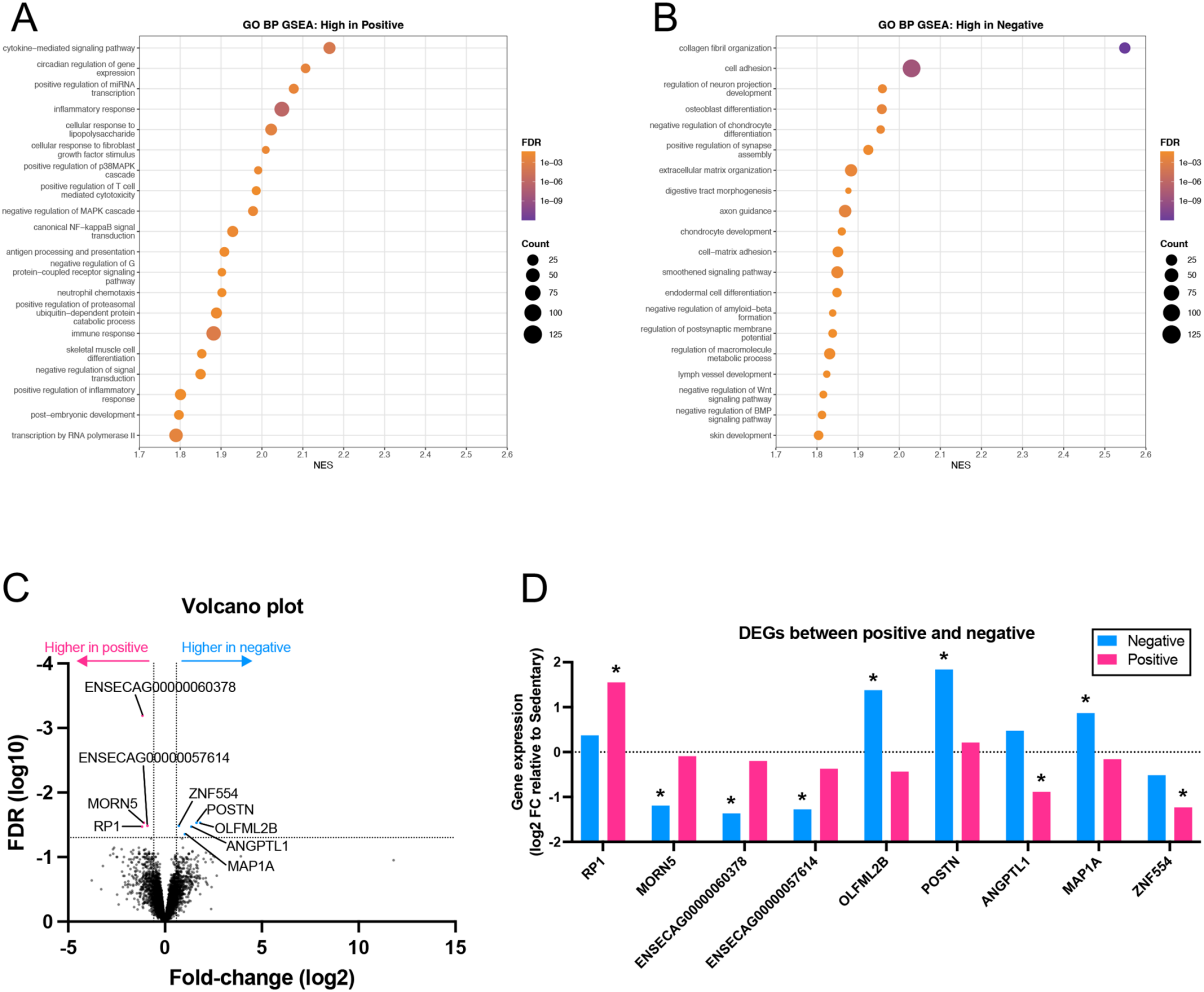
**Differential transcriptional and pathway responses between pacing strategies.** (A,B) GSEA of GO BP terms comparing positive and negative pacing strategies. Dot plots show the top enriched pathways among genes expressed at higher levels in the positive (A) and negative (B) pacing conditions. The x-axis represents normalized enrichment score (NES), and the y-axis indicates GO BP terms. Dot size reflects the number of core enriched genes (Count), and color indicates the false discovery rate (FDR). (C) Volcano plot illustrating differential gene expression between negative and positive pacing. The x-axis represents log_2_ fold change (negative versus positive), and the y-axis represents −log_10_ false discovery rate (FDR). Genes with higher expression in the positive pacing are shown on the left (pink), whereas genes with higher expression in the negative pacing are shown on the right (blue). Selected differentially expressed genes are annotated. (D) Log_2_ fold change in gene expression relative to the sedentary condition for genes that differed significantly in positive and negative pacing. Bars represent log_2_ fold change for positive (pink) and negative (blue) conditions relative to sedentary controls. An asterisk indicates a significant difference from the sedentary condition (FDR<0.05).

To identify biological processes associated with pacing-dependent differences, we performed gene set enrichment analysis (GSEA) using GO BP terms by directly comparing gene expression profiles between the two pacing strategies. Pathways related to immune and inflammatory responses were prominently enriched among genes with higher expression in the positive-pacing condition (Fig. 3A). These included cytokine-mediated signaling, regulation of gene expression, inflammatory response, neutrophil chemotaxis, and antigen processing and presentation. Conversely, genes with higher expression in the negative-pacing condition were significantly enriched for pathways associated with extracellular matrix (ECM) organization, tissue development, and cellular structural remodeling (Fig. 3B). These included collagen fibril organization, cell adhesion, ossification, chondrocyte development, and regulation of cell morphogenesis.

Consistent with the largely overlapping global transcriptomic responses, the direct comparison between pacing conditions identified only a small number of differentially expressed genes (Fig. 3C). Among the differentially expressed genes (DEGs), *RP1*, *MORN5*, and two unannotated Ensembl genes (*ENSECAG00000060378* and *ENSECAG00000057614*) were expressed at higher levels in the positive-pacing condition, whereas *OLFML2B*, *POSTN*, *ANGPTL1*, *MAP1A*, and *ZNF554* showed higher expression in the negative-pacing condition (Fig. 3D). Several genes with higher expression in the negative-pacing condition are associated with extracellular matrix organization or tissue remodeling, consistent with the pathway-level enrichment observed in Fig. 3B. Collectively, these findings indicate that the overall skeletal muscle transcriptomic response to high-intensity exercise was largely conserved between pacing strategies, with pacing-dependent differences restricted to a limited number of genes and pathways.

## DISCUSSION

### Shared core transcriptional responses between pacing strategies

The present study demonstrated that high-intensity exercise induced a pronounced and consistent transcriptional response in equine skeletal muscle, regardless of pacing pattern, characterized by large-scale changes in gene expression relative to the sedentary condition. PCA, hierarchical clustering, and Venn diagram analyses revealed that exercise per se, rather than the temporal distribution of exercise intensity, was the primary driver of global transcriptomic variation. These findings are consistent with previous reports showing that acute high-intensity exercise robustly induces transcriptional programs related to metabolic regulation, tissue remodeling, and stress adaptation in equine skeletal muscle (Bryan et al., 2017; Takahashi et al., 2025a,b,c). Among the genes commonly upregulated after both pacing strategies, several have also been implicated in skeletal muscle responses to exercise. For example, *NR4A3* is an exercise-responsive nuclear receptor associated with skeletal muscle metabolic regulation and oxidative phenotype (Pearen et al., 2012; Pillon et al., 2020). In addition, *IL6* and *LIF* encode contraction-responsive cytokines or myokines that have been linked to metabolic regulation and muscle remodeling (Broholm et al., 2011; Serrano et al., 2008). Thus, the temporal pattern of intensity distribution appeared to modulate limited aspects of gene regulation, without substantially altering the core transcriptomic response to high-intensity exercise.

### Transcriptional features associated with positive pacing

Although the overall transcriptional profiles largely overlapped between pacing strategies, positive pacing showed relative enrichment of immune- and inflammation-related pathways, including cytokine-mediated signaling. This may reflect greater acute physiological stress when the higher-intensity phase occurs at the beginning rather than at the end of exercise, despite identical total workload, consistent with our previous finding that positive pacing induced greater skeletal muscle glycogen depletion and glycolytic activation than negative pacing (Takahashi et al., 2024). Thus, our hypothesis that positive pacing would elicit a more pronounced post-exercise transcriptomic response was only partially supported, as positive pacing was associated with selected immune- and stress-related features but did not fundamentally alter the core transcriptomic response to high-intensity exercise.

Immune and inflammatory responses in skeletal muscle can have either adaptive or maladaptive consequences depending on their magnitude, duration, and physiological context. Chronic inflammation is associated with metabolic disorders such as type 2 diabetes (Pillon et al., 2022), whereas damaging contractions induce immune and inflammatory responses as part of muscle repair and remodeling (Peake et al., 2017), and transient activation of immune-related pathways after exercise may contribute to physiological remodeling (Nyasha et al., 2023). Therefore, it remains unclear whether the greater immune- and inflammation-related transcriptional responses observed after positive pacing reflect enhanced adaptive remodeling signals or excessive physiological strain. Genes expressed at higher levels after positive pacing included *RP1*, *MORN5*, and two unannotated equine transcripts. Because the functions of these genes in skeletal muscle are poorly characterized, their biological significance in the present study remains uncertain. Although *RP1* expression has previously been shown to be responsive to oxygen availability during exercise in equine skeletal muscle (Takahashi et al., 2025b), its function outside the retina remains unclear (Liu et al., 2004), and these limited gene-level differences should therefore be interpreted cautiously.

### Transcriptional features associated with negative pacing

Among the genes expressed at higher levels in the negative-pacing condition, several were related to extracellular matrix organization and structural remodeling, including *POSTN* and *MAP1A*. *POSTN* encodes periostin, a matricellular protein involved in extracellular matrix remodeling, and its upregulation has been linked to excessive extracellular matrix deposition and fibrosis in pathological contexts such as muscular dystrophy and severe muscle injury (Hara et al., 2018; Lorts et al., 2012). However, in the present acute exercise setting, higher *POSTN* expression alone should not be interpreted as evidence of pathological remodeling. Together with the enrichment of extracellular matrix-related pathways, the higher expression of *POSTN* and *MAP1A* may reflect limited pacing-dependent modulation of structural remodeling-related transcriptomic features, consistent with previous evidence that tissue remodeling and extracellular matrix adaptation are components of skeletal muscle responses to exercise training (Kritikaki et al., 2021; Pillon et al., 2020). In addition, the higher expression of *ANGPTL1* in the negative-pacing condition may suggest a potential link to matrix–vascular remodeling, given its reported roles in angiogenesis, vascular stability, and endothelial cell function (Santulli, 2014). Nevertheless, the functional significance of these negative-pacing-associated features remains to be established, particularly with respect to extracellular matrix structure, vascular remodeling during long-term adaptation.

### Implications for muscle adaptation and future directions

The limited number of DEGs between pacing strategies suggests that the skeletal muscle transcriptional response to high-intensity exercise was largely conserved between positive and negative pacing. Thus, exercise itself appeared to be the primary driver of post-exercise gene expression changes, whereas the temporal distribution of exercise intensity had a comparatively modest effect. From a training perspective, the present findings do not allow us to determine whether positive or negative pacing is more beneficial for recovery or long-term muscle remodeling. Positive pacing was associated with immune- and inflammation-related features, whereas negative pacing was associated with extracellular matrix- and tissue remodeling-related features. These differences may reflect distinct aspects of acute physiological stress or remodeling signals, but they do not establish the superiority of either pacing strategy. This aligns with physiological observations that different training protocols can produce similar overall metabolic demands while altering localized or temporal stress within muscle (Mohr et al., 2007). However, whether the modest transcriptomic differences observed here contribute to differences in recovery or long-term adaptation remains unknown. Given that training-induced skeletal muscle adaptations are thought to arise from repeated transient molecular responses to individual exercise bouts (Perry et al., 2010), training interventions will be needed to determine whether the modest acute changes observed here translate into physiologically relevant differences.

A limitation of the present study is the focus on a single post-exercise time point. Transcriptional responses to acute exercise are highly dynamic, and additional pacing-dependent differences may emerge at earlier or later time points (Kuang et al., 2022; Kusano et al., 2024). Another limitation is that possible sequence or carryover effects cannot be fully excluded, because a single pre-exercise biopsy collected before the first trial was used as the sedentary reference sample for both post-exercise conditions. However, the order of pacing conditions was randomized and balanced, and a washout period of at least six days was used to minimize potential order effects. In addition, because the present study used bulk RNA sequencing, we could not distinguish muscle fiber-intrinsic transcriptional changes from changes related to stromal, vascular, or immune cell activity. Functional validation of the identified genes is also required, particularly for genes with limited annotation in the equine genome.

### Conclusions

Acute high-intensity exercise induced a robust and largely conserved transcriptional response in equine skeletal muscle, regardless of exercise intensity distribution. Both positive and negative pacing strategies regulated overlapping gene networks related to metabolism, hypoxia signaling, and tissue remodeling, indicating that exercise itself is the primary driver of the transcriptomic response. Although a small number of genes differed between pacing strategies despite identical total workload, the biological significance of these modest pacing-dependent differences remains unknown. Further studies are needed to determine whether these differences have functional relevance for recovery, muscle remodeling, or long-term training adaptation.

## MATERIALS AND METHODS

### Animals and ethical approval

Eight Thoroughbred horses (three geldings and five females; age: 4–8 years; body weight: 515±60 kg; V̇O_2_max: 163±10 ml/kg/min) were used in this study. The experimental protocols were reviewed and approved by the Animal Welfare and Ethics Committee of the Japan Racing Association Equine Research Institute (approval number: 23-27). All experiments were performed and reported in accordance with our institutional guidelines and the ARRIVE guidelines. All incisions for catheter placement and muscle biopsies were made under local anesthesia using lidocaine. All efforts were made to minimize animal discomfort. The animals, exercise protocols, and cardiometabolic measurements employed in the present study have been described in detail in our previous publication (Takahashi et al., 2024). The current study represents an independent analysis focusing on skeletal muscle transcriptomic responses using RNA sequencing, based on muscle samples obtained from the same experimental sessions.

### Exercise testing and high-intensity exercise protocol

Incremental exercise testing for determining individual V̇O_2_max and the high-intensity exercise protocol were performed on a treadmill (Sato I, Sato AB, Uppsala, Sweden), as described previously (Kitaoka et al., 2011; Mukai et al., 2017). The horses wore an open-flow mask through which air was drawn by a rheostat-controlled blower. Airflow was measured using a pneumotachograph (LF-150B, Vise Medical, Chiba, Japan) connected to a differential pressure transducer (TF-5, Vise Medical, Chiba, Japan), and O_2_ and CO_2_ concentrations were measured using gas analyzers (FC-10 and CA-10, Sable Systems International, north Las Vegas, NV, USA).

One week after the incremental exercise test, horses completed two high-intensity exercise sessions in a randomized crossover design, separated by a washout period of at least 6 days. The order of the positive- and negative-pacing conditions was randomly assigned before the exercise trials, with balanced allocation such that four horses completed the positive-pacing condition first and four horses completed the negative-pacing condition first. This washout period was selected to minimize carryover effects between trials and exceeds the time required for recovery of muscle glycogen reported after glycogen-depleting exercise in horses (Lacombe et al., 2004). Exercise intensity was prescribed relative to each horse's V̇O_2_max. The 2-min bout was divided into two 1-min phases to manipulate the temporal distribution of exercise intensity while keeping total exercise duration and workload identical between pacing conditions. In the positive-pacing condition, horses exercised at 110% V̇O_2_max for the first 1 min, followed by 90% V̇O_2_max for the subsequent 1 min. In the negative-pacing condition, horses exercised at 90% V̇O_2_max for the first 1 min, followed by 110% V̇O_2_max for the subsequent 1 min. The treadmill speeds corresponding to 110% and 90% V̇O_2_max were 12.2 m/s (range: 11.2–12.6 m/s) and 10.0 m/s (range: 9.2–10.3 m/s), respectively. Each exercise session included a warm-up at 3.5 m/s for 3 min followed by 1.7 m/s for 1 min before the high-intensity exercise protocol, and a cool-down at 1.7 m/s for 10 min afterward.

### Muscle biopsy sampling

Muscle samples were collected prior to the first exercise trial and used as the pre-exercise reference samples for comparison with both post-exercise conditions. Additional muscle samples were collected at 4 h after each exercise session. The biopsy site was standardized at one-third of the distance from the coxal tuber along an imaginary line connecting the coxal tuber to the root of the tail. After shaving and aseptic preparation of the skin, local anesthesia was achieved by subcutaneous injection of 0.5 ml of 2% lidocaine (Sandoz K.K., Tokyo, Japan). Muscle biopsy samples were obtained from the middle gluteal muscle at a depth of approximately 5 cm using a 13-gauge×3.9 cm coaxial introducer needle and a 14-gauge×9 cm biopsy needle (SuperCore Biopsy Instrument, Argon Medical Devices, Plano, TX, USA). All muscle samples were immediately frozen in liquid nitrogen and stored at −80°C until further analysis.

### RNA extraction and sequencing

Total RNA was extracted from skeletal muscle tissues using the RNeasy Plus Universal Mini Kit (cat. no. 73404, Qiagen, Venlo, Limburg, The Netherlands) on the QIAcube Connect System (cat. no. 9002864, Qiagen). RNA quality and integrity were assessed using the Agilent RNA 6000 Nano Kit (cat. no. 5067-1511; Agilent Technologies) on an Agilent 5400 TapeStation system (Agilent Technologies), and only samples with adequate RNA integrity were used for library preparation. mRNA-seq libraries were constructed using the NEBNext Ultra II RNA Library Prep Kit for Illumina together with the NEBNext Poly(A) mRNA Magnetic Isolation Module (cat. nos E7770 and E7490, New England Biolabs, Ipswich, MA, USA), following the manufacturer's instructions. Library concentration and size distribution were evaluated using the Agilent DNA 7500 Kit (cat. no. 5067-1506, Agilent Technologies). Sequencing was performed by Novogene using the NovaSeq™ X Plus system (Illumina) with the NovaSeq X Series 10B Reagent Kit (300 cycles; cat. no. 20085594), generating 150-bp paired-end reads. FASTQ files were obtained from the sequencing provider, and initial quality control was performed using CLC Genomics Workbench version 25.0.1 (Qiagen) prior to downstream analyses.

### Bioinformatic and statistical analyses

Bioinformatic and statistical analyses were performed using integrated Differential Expression and Pathway analysis (iDEP; http://bioinformatics.sdstate.edu/idep96), GraphPad Prism software (version 10.1.1; GraphPad Software, La Jolla, CA, USA), and R (version 4.5.2). For RNA-seq data, raw gene-level read counts were normalized using the edgeR algorithm implemented in iDEP, with a minimum counts per million threshold of 0.5, pseudocount of 4, and gene median normalization.

PCA was performed using normalized gene expression values to visualize global transcriptomic differences among the pre-exercise baseline, positive-pacing, and negative-pacing conditions. For hierarchical clustering analysis, genes were ranked according to the variance of their normalized expression values across all samples, and the top 1000 most variable genes were selected. This number was chosen to visualize major transcriptomic variation across conditions while reducing the influence of low-variance genes that contribute limited information to clustering. Gene expression values were centered and scaled by gene before visualization.

Differentially expressed genes were identified using DESeq2 with a repeated-measures design formula: design=horse+condition. In this model, horse identity was included as a blocking factor to account for repeated measurements obtained from the same horse, whereas condition represented the experimental condition, including the pre-exercise baseline, positive-pacing, and negative-pacing samples. The baseline sample collected before the first exercise trial was used as the pre-exercise control for comparison with both post-exercise conditions. Pairwise contrasts were performed to compare positive pacing versus baseline, negative pacing versus baseline, and positive pacing versus negative pacing. Multiplicity was controlled using the Benjamini–Hochberg false discovery rate (FDR) procedure, and genes were considered differentially expressed when they met both criteria of FDR<0.05 and a minimum fold change of 1.5.

Venn diagram analysis was used to visualize the overlap of genes significantly upregulated or downregulated after positive and negative pacing compared with the baseline condition. Genes appearing in only one section of the Venn diagram were interpreted as genes that were significantly differentially expressed relative to baseline in one exercise condition but did not meet the differential expression criteria relative to baseline in the other condition. These genes were not interpreted as statistically significant pacing-dependent genes unless they were also identified in the direct positive- versus negative-pacing contrast.

GO biological process annotations were obtained from Ensembl using the biomaRt package in R. Over-representation analysis (ORA) and GSEA were performed using the clusterProfiler package in R to identify biological processes associated with differentially expressed genes and ranked gene expression changes, respectively. For ORA and GSEA, significance was assessed using FDR-adjusted *P*-values, with FDR<0.05 considered statistically significant.

To visualize pathway-level expression patterns across the three conditions, GO BP pathway activity scores were calculated using single-sample gene set enrichment analysis (ssGSEA) implemented in the GSVA package in R. Gene sets containing 10–500 genes were included. For each pathway, ssGSEA scores were compared across the pre-exercise baseline, positive-pacing, and negative-pacing conditions using one-way ANOVA, and multiplicity was controlled using the Benjamini–Hochberg FDR procedure. The top 20 pathways ranked by FDR were selected for heatmap visualization using the pheatmap package. Pathway scores were centered and scaled across samples for each pathway before visualization. Equus caballus–specific gene annotation databases were used to ensure accurate functional interpretation.

## Supplementary Material



10.1242/biolopen.062724_sup1 Supplementary information

Table S1. Gene ontology biological process (GOBP) gene set enrichment analysis (GSEA) among 3 groups.
