# Supplementary material for: Conserved skeletal muscle transcriptomic responses to pacing strategies in Thoroughbred horses
Source: Biol Open. 2026 Jul 17;15(7):bio062724. doi: 10.1242/bio.062724 (PMC13446558; doi:10.1242/bio.062724)
Supplement: Supplementary file 1 [file biolopen-15-062724-s1.pdf]

Table S1. Gene ontology biological process (GOBP) gene set enrichment analysis (GSEA) among 3 groups.

Available for download at
https://journals.biologists.com/bio/article-lookup/doi/10.1242/bio.062724#supplementary-data

Biology Open • Supplementary information
