# Supplementary material for: Conserved skeletal muscle transcriptomic responses to pacing strategies in Thoroughbred horses
Source: Biol Open. 2026 Jul 17;15(7):bio062724. doi: 10.1242/bio.062724 (PMC13446558; doi:10.1242/bio.062724)
Supplement: Supplementary file 2 [file biolopen-15-062724_review_history.pdf]

Conserved skeletal muscle transcriptomic responses to pacing strategies in Thoroughbred horses

Kenya Takahashi, Kazutaka Mukai, Takanaga Shirai, Yusaku Ebisuda, Fumi Sugiyama, Toshinobu Yoshida, Hideo Hatta and Yu Kitaoka

10.1242/bio.062724

Editor: Valentina di Santo

Review timeline

Original submission:	31st May 2026
Editorial decision:	6 June 2026
First revision received:	21 June 2026
Accepted:	26th June 2026

Original submission

First decision letter

MS ID#: bio.062724

MS Title: Conserved skeletal muscle transcriptomic responses to pacing strategies in Thoroughbred horses

Authors: Kenya Takahashi, Kazutaka Mukai, Takanaga Shirai, Yusaku Ebisuda, Fumi Sugiyama, Toshinobu Yoshida, Hideo Hatta and Yu Kitaoka

I have now reached a decision on the above manuscript.

The reviewer reports are shown at the bottom of this email.

As you will see, the reviewers raised a number of substantial criticisms that prevent me from accepting the paper at this stage.

They suggest, however, that a revised version might prove acceptable, if you can address their concerns. If you think that you can deal satisfactorily with the criticisms on revision, I would be pleased to see a revised manuscript. We would then return it to the reviewers.

At this stage, we also ask you to ensure your manuscript complies with our formatting guidelines. Provided you are able to fully address the referees' comments, we are positive about publication of your paper (we accept over 95% of revision submissions) and therefore hope you won't mind any extra work involved in reformatting your manuscript at this point.

Please upload both a 'clean' version of your Word file, along with a highlighted version clearly showing where you have made changes in the revised manuscript. Please avoid using 'Track changes' in Word files as these are lost in PDF conversion.

I should be grateful if you would also provide a point-by-point response detailing how you have dealt with the points raised by the reviewers in the 'Response to Reviewers' box. Please attend to all of the reviewers' comments. If you do not agree with any of their criticisms or suggestions please explain clearly why this is so.

© 2026. Published by The Company of Biologists under the terms of the Creative Commons Attribution License (https://creativecommons.org/licenses/by/4.0/).

Reviewer 1

Comments for the author

The authors present a well-written study into the genetic activity of skeletal muscle in response to pacing strategy. While I have no major concerns about the manuscript, I think that a few additional details and possibly reframing the results could improve it. Specific comments are below:

- I feel there is an overemphasis on the "fine-tuning of the expression of a limited subset of genes" here. Ultimately, there are only 9 genes that are differentially expressed due to pacing strategy, and multiple thousands that are the same. Should the major conclusions therefore not be "expression of genes is remarkably conserved between pacing strategies"? It feels like the authors are trying too hard to find significant differences when they don't necessarily exist, particularly when two of the four genes which are positively expressed after positive pacing have unknown functions.

- Related to this, it may be an improvement to include a hypothesis at the end of the Introduction (line 71), stating what you thought might happen in terms of gene regulation in response to different pacing, and why (citing relevant literature). This hypothesis can then be confirmed/rejected in the Discussion. This could make it slightly easier to discuss a negative result (i.e. the conservation of gene expression), if you hypothesised that the opposite would happen, for example.

- Line 70- change "4 h" to "four hours"
- Line 71- change "2-min" to "two minutes"

- I appreciate that the experimental methods used here are the same as previous studies, but I think a few more details need to be added here. Specifically, what equipment/method was used to estimate VO2 max from each horse? What kind of treadmill was used (Make/model)?

- What gait were the horses using (I assume galloping, but "ran" isn't really an appropriate term for quadrupedal gait)?

What were the average speeds of the positive and negative pacing periods?

- Are there justifications for the timings used? I.e.. the 1 min periods of exercise, the 6 day washout period, and the 4-hour post-exercise biopsy collection.

- Why were biopsies not collected prior to the second exercise session, to confirm that gene expression had returned to baseline?

- In the Discussion, it is mentioned that "subtle transcriptomic modulation may contribute to differences in recovery or long-term adaptation". If you are going to stick with the conclusion that these subtle differences are important, then do you have any thoughts on which pacing strategy is better for recovery or muscle remodelling? It would make a nice ending to the paper to state the advantages/disadvantages of particular pacing strategies.

- Figure 3- change "Volucano" to "Volcano". Add a legend to D, showing which bar refers to which condition (I know it's in the caption, but it should be in the figure itself, too).

Reviewer 2

Comments for the author

The authors have conducted a trial whereby muscle transcriptomic responses were measured in 8 thoroughbred horses that underwent two volume-matched high-intensity sprint sessions, with one starting at a higher intensity that subsequently reduced, while in the other the reverse occurred (defined as positive and negative pacing). They reported broadly similar transcriptomic responses to both high-intensity sessions compared to a rested control sample, with some differences

© 2026. Published by The Company of Biologists under the terms of the Creative Commons Attribution License (https://creativecommons.org/licenses/by/4.0/).

between the positively and negatively paced sessions, potentially indicating that pacing patterns subtly alters specific gene expression.

Overall, I found this article interesting and believe it is a useful and important research question. Certain aspects of the manuscript, and particularly the statistical analysis, were, however, unclear, which made it difficult to fully understand both the analyses undertaken and in turn, results interpretation. I suggest that some clarification and revision would be beneficial to the manuscript, mainly focusing on a more explicit justification of the research question and important design elements, along with a more detailed and clearer explanation of the statistical analysis. Below, I have made a number of comments, in the order that they appear in the manuscript, which I hope will be of use to the authors.

Specific comments:

1. I suggest that a more in-depth theoretical justification for the study would be useful, particularly a more explicit elaboration of the precise research question under investigation. For example, biopsy timing is particularly relevant in studies like this, considering that exercise induced gene expression are highly dynamic. Different biological pathways peak at different times, meaning that a single biopsy time-point provides only a partial snapshot of the molecular response. As such, it would be useful to elaborate on what aspect of the transcriptional response you were primarily interested in evaluating, to gauge whether this could be detected in a biopsy collected 4-hours post-exercise. To be clear, I do not disagree with the timing, and recognize that a brief comment was made on this point later in the manuscript (lines 322 - 325), however as this underpins the entire study, I would expect the timing and its relevance to be discussed and justified earlier in the manuscript.

2. Line 62 - 64: While I certainly do not disagree with the use of thoroughbred horses as a model, I was interested in the statement that"owing to their exceptional exercise capacity, thoroughbreds provide a controlled system in which exercise intensity can be precisely prescribed". What is it about their exercise capacity that influences the precision with which exercise intensity can be prescribed? Arguably, intensity can be just as precisely prescribed in human models, considering the potential for two-way verbal feedback to ensure that protocols are strictly adhered to, along with the capacity for live-time and simultaneous monitoring of multiple exercise response variables.

3. Line 66 - 70: Please provide further details on the findings from the previous study mentioned here. The phrasing, and particularly the statement that the goal of this study was "to determine whether these physiological differences are accompanied by distinct transcriptional responses"implies that the present study was hypothesis driven and informed by previous findings. If this is the case, the specific hypotheses should be clearly articulated. In contrast, line 84 states that this was an independent study, implying that this study was primarily considered as an exploratory untargeted analysis. If this is the case, this should also be explicitly stated.

4. Further explanation and justification for these timing decisions would be useful. Why was a 2-minute exercise bout selected? As described above, it would also be interesting to explain the rationale for the biopsy timing, and to be more explicit about what aspects of the transcriptional response this biopsy is best suited to assess, along with what aspects it cannot capture. Taking the biopsy 4-hour post-exercise bout makes sense if the goal is to primarily focus on secondary transcriptional programs and adaptive responses, but less so if the focus is early signaling pathways, or later repair or recovery related processes. This points links into point 3 above. If there were specific hypotheses underpinning this study, these should be made clear, as understanding the rationale underpinning the biopsy timing will be fundamental to knowing whether the hypothesized responses are likely to be captured.

5. Line 100: When exactly was the control biopsy taken? Was the sample collected prior to the first exercise trial the control against which both exercise responses were assessed, or was each high-intensity exercise session compared against a control sample collected under matched conditions? This has important implications for interpretation, considering the potential for increased biological variation over time.

© 2026. Published by The Company of Biologists under the terms of the Creative Commons Attribution License (https://creativecommons.org/licenses/by/4.0/).

6. Please provide further information on the cross-over design. How was randomization conducted? Was order allocation balanced? Were sequence effects considered within the statistical analysis? This information is particularly relevant considering the sample size of 8 horses and if I have understood correctly, the use of a single baseline to compare both high-intensity exercise conditions.

7. Line 127 - 141: I found the statistics section somewhat difficult to follow, and some details that may be important for data interpretation were either unclear or missing. Furthermore, a number of analyses presented in the results section were not explained in the statistics section, e.g., the principal component analysis, the Venn diagram analysis and some of the pathway-level analyses. I acknowledge that I am not a biostatistician and may have misunderstood some aspects of the methodology employed, however it is likely that this paper will be of particular interest to exercise and muscle physiologists and I believe it is important that the statistical analysis is clearly and completely explained. Specific points to consider include:

- Please explain how the repeated-measures crossover design was incorporated into the analysis and particularly how the baseline samples were handled. Providing the exact design formula used for differential expression analysis would be very useful.

- Please clarify how repeated measurements from the same horse were accounted for within the DESeq2 design formula? This may have important implications considering the likely strong correlation between the repeated samples obtained from each horse.

- At multiple times throughout the paper different "groups" were referred to, but I suggest that you instead refer to different conditions, considering that all analyses were conducted on repeated measures from the same group.

- Line 132: How were the top 1,000 more variable genes selected for the hierarchical clustering analysis?

- How was multiplicity handled throughout the analysis? Line 140 states that differential expression analysis was performed using DESeq2 with an FDR of < 0.05 and a minimum fold change of 1.5, however directly afterwards the manuscript states that statistical significance was defined as $p < 0.05$. Please clarify whether reported p-values are adjusted or unadjusted and exactly what significance criteria were applied to the different analyses.

- Further to the above point, please clarify exactly how differences and similarities were identified, which will include clarification on the control condition and how it was incorporated within the model. For example, lines 172 - 176 report large number of genes differentially expressed in each exercise condition relative to control, but only 9 genes differentially expressed between pacing conditions. I understand that different analysis approaches can lead to different outcomes, but a clearer understanding of the criteria by which differences and similarities were assessed would be helpful to guide interpretation of these findings.

- The interpretation of "condition-specific" genes based on the Venn diagram analysis was also somewhat unclear. From what I understand, this analysis represents genes that were differentially expressed in one exercise-versus-control comparison, but this does not necessarily imply a statistically significant difference between pacing conditions.

8) The current discussion largely focuses on the observed differences between the pacing strategies. While the broad conclusion of the a largely conserved transcriptional response, with some potential modulated expression of limited genes seems reasonable based on the reported data, I do suggest caution throughout in interpreting individual gene differences, considering the large potential for biological variation in studies of this kind. Greater clarity on the statistical approach employed, and particularly the points described above will be very useful in better understanding the discussion. It is possible that some points may require some reconsideration depending on the authors response to the above points.

Reviewer's Responses to Questions

© 2026. Published by The Company of Biologists under the terms of the Creative Commons Attribution License (https://creativecommons.org/licenses/by/4.0/).

Experimental quality
Are experiments performed using appropriate methods that will answer the question (or test the hypothesis or support the observations) posed by the authors? Is the right tool used for the job?
If 'No', please indicate reasons in Comments for Author box below.
Reviewer #1:

- Yes

Reviewer #2:

- Yes

Were the data analyzed using appropriate statistical tests?
If 'No', please indicate reasons in Comments for Author box below.
Reviewer #1:

- Yes

Reviewer #2:

- Yes

Reproducibility
Were experiments performed using adequate number of biological replicates?
If 'No', please indicate reasons in Comments for Author box below.
Reviewer #1:

- Yes

Reviewer #2:

- Yes

Is there sufficient raw data to assess the rigor of the analysis?
If 'No', please indicate reasons in Comments for Author box below.
Reviewer #1:

- Yes

Reviewer #2:

- Yes

Does the methods section provide sufficient detail to permit reproducibility?
If 'No', please indicate reasons in Comments for Author box below.
Reviewer #1:

- No

Reviewer #2:

- No

Completeness
Are the manuscript's conclusions supported by the data?
If 'No', please indicate reasons in Comments for Author box below.
Reviewer #1:

- No

Reviewer #2:

- Yes

© 2026. Published by The Company of Biologists under the terms of the Creative Commons Attribution License (https://creativecommons.org/licenses/by/4.0/).

Scholarship
Do the authors cite and discuss the merits of data that would argue for and against their conclusion?
If 'No', please indicate reasons in Comments for Author box below.
Reviewer #1:

- Yes

Reviewer #2:

- Yes

Does the manuscript title & abstract accurately reflect the contents of the manuscript, without hyperbole?
If 'No', please indicate reasons in Comments for Author box below.
Reviewer #1:

- Yes

Reviewer #2:

- Yes

First revision

Author response to reviewers' comments

Dear Editor and Reviewers,

We thank the Editor and both reviewers for their careful evaluation of our manuscript and for their constructive comments. We are pleased that the reviewers found the study interesting and potentially useful. In response to the comments, we have revised the manuscript to emphasize more clearly that the skeletal muscle transcriptional response to high-intensity exercise was largely conserved between pacing strategies, with only limited pacing-dependent differences. We have also added further methodological details, clarified the experimental design and statistical analyses, and tempered the interpretation of individual gene-level differences.

Comments from the Reviewers:

Reviewer 1:

The authors present a well-written study into the genetic activity of skeletal muscle in response to pacing strategy. While I have no major concerns about the manuscript, I think that a few additional details and possibly reframing the results could improve it. Specific comments are below:

Our response:
We would like to thank the reviewer for the positive assessment of our manuscript and for the constructive comments. We have carefully considered these suggestions and have revised the manuscript accordingly. In particular, we have added methodological details and reframed the results to emphasize that the skeletal muscle transcriptomic response was largely conserved between pacing strategies, with only limited pacing-dependent differences. The following is our point-by-point responses to each of the comments. The changes in the revised manuscript are highlighted.

- I feel there is an overemphasis on the "fine-tuning of the expression of a limited subset of genes" here. Ultimately, there are only 9 genes that are differentially expressed due to pacing strategy, and multiple thousands that are the same. Should the major conclusions therefore not be "expression of genes is remarkably conserved between pacing strategies"? It feels like the authors

© 2026. Published by The Company of Biologists under the terms of the Creative Commons Attribution License (https://creativecommons.org/licenses/by/4.0/).

are trying too hard to find significant differences when they don't necessarily exist, particularly when two of the four genes which are positively expressed after positive pacing have unknown functions.

Our response:
We agree that the original manuscript may have overemphasized the small number of pacing-dependent differentially expressed genes. The direct comparison between pacing strategies identified only nine differentially expressed genes, whereas the vast majority of exercise-responsive genes were shared between positive and negative pacing. We have therefore revised the Abstract, Results, Discussion, and Conclusions to emphasize that exercise itself was the primary driver of the skeletal muscle transcriptomic response, which was largely conserved between pacing strategies. We have also tempered the language related to "fine-tuning" and clarified that the biological significance of the limited pacing-dependent differences remains uncertain. To reflect this revised emphasis, we have changed the title to "Conserved skeletal muscle transcriptomic responses to pacing strategies in Thoroughbred horses".

- Related to this, it may be an improvement to include a hypothesis at the end of the Introduction (line 71), stating what you thought might happen in terms of gene regulation in response to different pacing, and why (citing relevant literature). This hypothesis can then be confirmed/rejected in the Discussion. This could make it slightly easier to discuss a negative result (i.e. the conservation of gene expression), if you hypothesised that the opposite would happen, for example.

Our response:
We agree with the reviewer's suggestion to include a hypothesis at the end of the Introduction. We have revised the Introduction to state that, based on our previous finding that positive pacing induced greater glycogen depletion than negative pacing during workload-matched high-intensity exercise, we hypothesized that positive pacing would elicit a more pronounced post-exercise transcriptomic response in equine skeletal muscle during early recovery. We have also addressed this hypothesis in the Discussion by noting that it was only partially supported: although positive pacing showed modest enrichment of immune- and inflammation-related pathways and a small number of pacing-dependent differentially expressed genes, the overall transcriptomic response was largely conserved between pacing strategies.

- Line 70- change "4 h" to "four hours"
- Line 71- change "2-min" to "two minutes"

Our response:
We have made the suggested changes accordingly.

- I appreciate that the experimental methods used here are the same as previous studies, but I think a few more details need to be added here. Specifically, what equipment/method was used to estimate VO2 max from each horse? What kind of treadmill was used (Make/model)?

Our response:
We have revised the Methods section to describe the equipment and procedures used to determine individual VO_2max and to specify the treadmill make and model. We now state that the incremental exercise test and high-intensity exercise protocols were performed on a treadmill (Sato I, Sato AB, Uppsala, Sweden). We also added that, during incremental exercise testing, horses wore an open-flow mask through which air was drawn by a rheostat-controlled blower; airflow was measured using a pneumotachograph (LF-150B, Vise Medical, Chiba, Japan) connected to a differential pressure transducer (TF-5, Vise Medical, Chiba, Japan); O_2 and CO_2 concentrations were measured using gas analyzers (FC-10 and CA-10, Sable Systems International, North Las Vegas, NV, USA).

- What gait were the horses using (I assume galloping, but "ran" isn't really an appropriate term for quadrupedal gait)?

Our response:

© 2026. Published by The Company of Biologists under the terms of the Creative Commons Attribution License (https://creativecommons.org/licenses/by/4.0/).

We agree that "ran" is not the most appropriate term for describing quadrupedal locomotion. The exercise intensity in the present study was prescribed based on treadmill speed corresponding to each horse's individual $\dot{V}O_2max$, rather than by formal gait classification. Although the horses were likely galloping during the 110% $\dot{V}O_2max$ phase, gait was not systematically recorded for each horse and each intensity phase, and some horses may have used a canter during the 90% $\dot{V}O_2max$ phase. Therefore, we have revised the manuscript to avoid the term "ran" and have used more neutral wording such as "exercised" or "performed treadmill exercise" throughout the manuscript.

- What were the average speeds of the positive and negative pacing periods?

Our response:
We have revised the Methods section to include the average treadmill speeds corresponding to the 110% and 90% $\dot{V}O_2max$ intensities, together with their ranges. Specifically, we now report that the treadmill speeds corresponding to 110% and 90% $\dot{V}O_2max$ were 12.2 m/s (range: 11.2–12.6 m/s) and 10.0 m/s (range: 9.2–10.3 m/s), respectively.

- Are there justifications for the timings used? I.e.. the 1 min periods of exercise, the 6 day washout period, and the 4-hour post-exercise biopsy collection.

Our response:
We thank the reviewer for raising this important point. We have revised the Introduction and Methods sections to clarify the rationale for the timing used in the study. The two-minute high-intensity exercise bout was selected to approximate the time scale of Thoroughbred racing, as many such race events are completed within approximately two minutes. This duration was considered physiologically relevant given that previous studies have demonstrated significant metabolic changes in the skeletal muscle of Thoroughbreds (Harris et al., 1987; Kitaoka et al., 2014). Within this bout, exercise was divided into two one-minute phases based on our previously established workload-matched pacing protocol (Takahashi et al., 2024), allowing us to manipulate the temporal distribution of exercise intensity while keeping total exercise duration and workload identical between pacing conditions. Specifically, the order of the 110% and 90% $\dot{V}O_2max$ phases was reversed between the positive- and negative-pacing conditions. We also clarified the rationale for the washout period and biopsy timing. The washout period of at least six days was selected to minimize potential carryover effects between the two high-intensity exercise trials. This interval was consistent with our previous high-intensity exercise studies in Thoroughbred horses using crossover designs (Takahashi et al., 2024; Takahashi et al., 2025a; Takahashi et al., 2025b) and was longer than the 72-hour period over which muscle glycogen repletion has been reported after glycogen-depleting exercise in horses (Lacombe et al., 2004). The four-hour post-exercise biopsy time point was selected because previous equine studies have shown robust skeletal muscle gene expression changes at this stage after exercise (McGivney et al., 2009; Bryan et al., 2017), and human studies also support the period approximately 3–4 hours after endurance or high-intensity exercise as a key window for detecting mRNA responses (Popov et al., 2019; Kuang et al., 2022). In addition, we acknowledge in the Discussion that residual sequence or carryover effects cannot be fully excluded, although the randomized and balanced crossover design together with the washout period was used to minimize these effects.

- Why were biopsies not collected prior to the second exercise session, to confirm that gene expression had returned to baseline?

Our response:
We acknowledge that collecting an additional pre-exercise biopsy before the second exercise session would have allowed us to directly confirm whether gene expression had returned to the sedentary baseline. In the present study, the pre-exercise biopsy collected before the first trial was used as the sedentary reference sample for both post-exercise conditions. A separate pre-exercise biopsy was not collected before the second exercise session in order to minimize the total number of invasive muscle biopsies for animal welfare reasons. We have clarified this point in the Methods section and added it as a limitation in the Discussion. Although residual sequence or carryover effects cannot be fully excluded, the order of pacing conditions was randomized and balanced, and a washout period of at least six days was used to minimize these effects. This approach is consistent with our previous high-intensity exercise studies in Thoroughbred horses using similar

© 2026. Published by The Company of Biologists under the terms of the Creative Commons Attribution License (https://creativecommons.org/licenses/by/4.0/).

crossover designs (Takahashi et al., 2025a; Takahashi et al., 2025b; Takahashi et al., 2025c). Importantly, the direct comparison between positive and negative pacing was performed within the repeated-measures crossover design and remains the most relevant analysis for evaluating pacing-dependent differences.

- In the Discussion, it is mentioned that "subtle transcriptomic modulation may contribute to differences in recovery or long-term adaptation". If you are going to stick with the conclusion that these subtle differences are important, then do you have any thoughts on which pacing strategy is better for recovery or muscle remodelling? It would make a nice ending to the paper to state the advantages/disadvantages of particular pacing strategies.

Our response:
We appreciate the reviewer's suggestion. In revising the Discussion, we have clarified that the present data do not allow us to determine whether positive or negative pacing is more advantageous for recovery or long-term muscle remodeling. We agree that the original wording may have implied that the subtle transcriptomic differences were functionally important, and we have therefore tempered this interpretation. The revised Discussion now states that positive pacing was associated with immune- and inflammation-related features, whereas negative pacing was associated with extracellular matrix- and tissue remodeling-related features; however, these differences do not establish the superiority of either pacing strategy. We have also added that repeated-training studies will be needed to determine whether the modest acute differences observed here translate into physiologically relevant differences.

- Figure 3- change "Volucano" to "Volcano". Add a legend to D, showing which bar refers to which condition (I know it's in the caption, but it should be in the figure itself, too).

Our response:
We have corrected the spelling of "Volcano" in Fig. 3 and added a legend to panel D indicating the corresponding conditions. During revision, we also noticed that the numbers of genes with higher expression in the positive- and negative-pacing conditions were mistakenly shown in reverse order in Fig. 2A. We have corrected this error so that the figure now accurately shows four genes with higher expression in the positive-pacing condition and five genes with higher expression in the negative-pacing condition.

Reviewer 2:

The authors have conducted a trial whereby muscle transcriptomic responses were measured in 8 thoroughbred horses that underwent two volume-matched high-intensity sprint sessions, with one starting at a higher intensity that subsequently reduced, while in the other the reverse occurred (defined as positive and negative pacing). They reported broadly similar transcriptomic responses to both high-intensity sessions compared to a rested control sample, with some differences between the positively and negatively paced sessions, potentially indicating that pacing patterns subtly alters specific gene expression.

Overall, I found this article interesting and believe it is a useful and important research question. Certain aspects of the manuscript, and particularly the statistical analysis, were, however, unclear, which made it difficult to fully understand both the analyses undertaken and in turn, results interpretation. I suggest that some clarification and revision would be beneficial to the manuscript, mainly focusing on a more explicit justification of the research question and important design elements, along with a more detailed and clearer explanation of the statistical analysis. Below, I have made a number of comments, in the order that they appear in the manuscript, which I hope will be of use to the authors.

Our response:
We would like to thank the reviewer for the careful reading of our manuscript and the detailed suggestions for improving its clarity and interpretation. We have revised the manuscript to make the research question and hypothesis more explicit, provide additional methodological and statistical details, and present a more balanced interpretation of the results. Specifically, we have expanded the Introduction and Methods sections, described the crossover design and use of the sedentary reference sample in greater detail, provided a fuller explanation of the differential

© 2026. Published by The Company of Biologists under the terms of the Creative Commons Attribution License (https://creativecommons.org/licenses/by/4.0/).

expression and pathway analyses, and reframed the Results and Discussion to emphasize the largely conserved transcriptomic response between pacing strategies while tempering the interpretation of the limited pacing-dependent gene-level differences. Below, we provide point-by-point responses to each comment.

Specific comments

1. I suggest that a more in-depth theoretical justification for the study would be useful, particularly a more explicit elaboration of the precise research question under investigation. For example, biopsy timing is particularly relevant in studies like this, considering that exercise induced gene expression are highly dynamic. Different biological pathways peak at different times, meaning that a single biopsy time-point provides only a partial snapshot of the molecular response. As such, it would be useful to elaborate on what aspect of the transcriptional response you were primarily interested in evaluating, to gauge whether this could be detected in a biopsy collected 4-hours post-exercise. To be clear, I do not disagree with the timing, and recognize that a brief comment was made on this point later in the manuscript (lines 322 - 325), however as this underpins the entire study, I would expect the timing and its relevance to be discussed and justified earlier in the manuscript.

Our response:
We appreciate this important comment. We have revised the Introduction to provide a more explicit theoretical justification for the study and to clarify the specific aspect of the transcriptomic response that we aimed to evaluate. In the revised manuscript, we now state that the study was designed to examine whether the temporal distribution of exercise intensity during a workload-matched high-intensity exercise bout influences the early recovery-phase skeletal muscle transcriptomic response. We have also added a clearer hypothesis based on our previous metabolomic findings that positive pacing induced greater glycogen depletion and glycolytic activation than negative pacing during workload-matched high-intensity exercise (Takahashi et al., 2024). We have also moved and expanded the justification for the four-hour biopsy time point earlier in the manuscript. Specifically, we now explain in the Introduction that this time point was selected because previous equine studies have shown robust skeletal muscle gene expression changes four hours after exercise (McGivney et al., 2009; Bryan et al., 2017), and human studies also support the period approximately 3–4 hours after endurance or high-intensity exercise as a key window for detecting mRNA responses (Popov et al., 2019; Kuang et al., 2022). We agree that a single biopsy time point provides only a partial snapshot of the dynamic molecular response to exercise, and we have retained this point as a limitation in the Discussion, noting that additional pacing-dependent differences may emerge at earlier or later recovery time points.

2. Line 62 - 64: While I certainly do not disagree with the use of thoroughbred horses as a model, I was interested in the statement that"owing to their exceptional exercise capacity, thoroughbreds provide a controlled system in which exercise intensity can be precisely prescribed". What is it about their exercise capacity that influences the precision with which exercise intensity can be prescribed? Arguably, intensity can be just as precisely prescribed in human models, considering the potential for two-way verbal feedback to ensure that protocols are strictly adhered to, along with the capacity for live-time and simultaneous monitoring of multiple exercise response variables.

Our response:
We thank the reviewer for this thoughtful comment. We agree that the original wording may have overstated the link between the exceptional exercise capacity of Thoroughbred horses and the precision with which exercise intensity can be prescribed. Our intention was not to imply that exercise intensity cannot be precisely prescribed in human studies, but rather to emphasize that Thoroughbred horses provide a large-animal model in which high-intensity exercise can be imposed under highly controlled treadmill conditions, with exercise intensity prescribed relative to each individual’s VO_2max. We have therefore revised the Introduction to describe this rationale more accurately and to avoid implying superiority over human exercise models.

3. Line 66 - 70: Please provide further details on the findings from the previous study mentioned here. The phrasing, and particularly the statement that the goal of this study was "to determine whether these physiological differences are accompanied by distinct transcriptional

© 2026. Published by The Company of Biologists under the terms of the Creative Commons Attribution License (https://creativecommons.org/licenses/by/4.0/).

responses"implies that the present study was hypothesis driven and informed by previous findings. If this is the case, the specific hypotheses should be clearly articulated. In contrast, line 84 states that this was an independent study, implying that this study was primarily considered as an exploratory untargeted analysis. If this is the case, this should also be explicitly stated.

Our response:

This comment helped us recognize that the original wording did not clearly distinguish between the hypothesis-driven rationale derived from our previous metabolomic study and the untargeted transcriptomic approach used in the present RNA-sequencing analysis. We have revised the Introduction to provide more specific details of the previous findings and to articulate the hypothesis more explicitly. Specifically, we now state that our previous metabolomic analysis showed that positive pacing induced greater glycogen depletion and glycolytic activation than negative pacing during workload-matched high-intensity exercise (Takahashi et al., 2024). Based on these findings, we hypothesized that positive pacing would elicit a more pronounced post-exercise transcriptomic response in equine skeletal muscle during early recovery. We have also revised the wording regarding the relationship between the previous and present studies. Our intention was not to imply that the present study was unrelated to the previous physiological and metabolomic findings, but rather that the RNA-sequencing analysis addressed a distinct research question using skeletal muscle samples collected during the same experimental sessions. We have therefore clarified that the present study was informed by the previous metabolomic findings, while using an untargeted transcriptomic approach to examine whether workload-matched pacing strategies differed in their post-exercise skeletal muscle gene expression profiles. In the Discussion, we now note that the hypothesis was only partially supported, because positive pacing showed modest enrichment of immune- and inflammation-related features, whereas the overall transcriptomic response was largely conserved between pacing strategies.

4. Further explanation and justification for these timing decisions would be useful. Why was a 2-minute exercise bout selected? As described above, it would also be interesting to explain the rationale for the biopsy timing, and to be more explicit about what aspects of the transcriptional response this biopsy is best suited to assess, along with what aspects it cannot capture. Taking the biopsy 4-hour post-exercise bout makes sense if the goal is to primarily focus on secondary transcriptional programs and adaptive responses, but less so if the focus is early signaling pathways, or later repair or recovery related processes. This points links into point 3 above. If there were specific hypotheses underpinning this study, these should be made clear, as understanding the rationale underpinning the biopsy timing will be fundamental to knowing whether the hypothesized responses are likely to be captured.

Our response:

We have addressed this point by expanding the rationale for both the exercise duration and the biopsy timing in the revised manuscript. The two-minute high-intensity exercise bout was selected to approximate the time scale of Thoroughbred racing, as many such race events are completed within approximately two minutes. Within this bout, exercise was divided into two one-minute phases based on our previously established workload-matched pacing protocol (Takahashi et al., 2024), allowing us to manipulate the temporal distribution of exercise intensity while keeping total exercise duration and workload identical between pacing conditions. We have also added references supporting that one to two minutes of high-intensity exercise is sufficient to induce marked metabolic perturbations in Thoroughbred skeletal muscle (Harris et al., 1987; Kitaoka et al., 2014). Moreover, we have clarified the rationale for the four-hour post-exercise biopsy time point. As noted in response to point 3, our primary interest was the early recovery-phase skeletal muscle transcriptomic response to workload-matched pacing strategies. We now explain in the Introduction that the four-hour time point was selected because previous equine studies have shown robust skeletal muscle gene expression changes at this stage after exercise (McGivney et al., 2009; Bryan et al., 2017), and human studies also support the period approximately 3–4 hours after endurance or high-intensity exercise as a key window for detecting mRNA responses (Popov et al., 2019; Kuang et al., 2022). We have also revised the Discussion to acknowledge that a single four-hour biopsy provides only a partial snapshot of the dynamic molecular response to exercise and may not capture very early signaling events or later recovery-related processes.

5. Line 100: When exactly was the control biopsy taken? Was the sample collected prior to the first exercise trial the control against which both exercise responses were assessed, or was each high-

© 2026. Published by The Company of Biologists under the terms of the Creative Commons Attribution License (https://creativecommons.org/licenses/by/4.0/).

intensity exercise session compared against a control sample collected under matched conditions? This has important implications for interpretation, considering the potential for increased biological variation over time.

Our response:
We have clarified in the revised Methods section that the pre-exercise biopsy was collected before the first exercise trial and was used as the sedentary reference sample for both post-exercise conditions. A separate pre-exercise biopsy was not collected before the second exercise session in order to minimize the total number of invasive muscle biopsies for animal welfare reasons. This approach is consistent with our previous RNA-sequencing studies in Thoroughbred horses using similar high-intensity exercise and crossover designs (Takahashi et al., 2025a; Takahashi et al., 2025b; Takahashi et al., 2025c). We agree that this design should be considered when interpreting the exercise-versus-sedentary comparisons, because a matched pre-exercise biopsy was not collected before each high-intensity exercise session. We have therefore added this point as a limitation in the Discussion, noting that residual sequence, carryover, or time-related variation cannot be fully excluded. Nevertheless, the order of pacing conditions was randomized and balanced, a washout period of at least six days was used to minimize carryover effects, and the direct comparison between positive and negative pacing was performed within the repeated-measures crossover design.

6. Please provide further information on the cross-over design. How was randomization conducted? Was order allocation balanced? Were sequence effects considered within the statistical analysis? This information is particularly relevant considering the sample size of 8 horses and if I have understood correctly, the use of a single baseline to compare both high-intensity exercise conditions.

Our response:
We have expanded the Methods section to provide further details on the randomized crossover design. We now state that the order of the positive- and negative-pacing conditions was randomly assigned before the exercise trials, with balanced allocation such that four horses completed the positive-pacing condition first and four horses completed the negative-pacing condition first. We have also clarified that the pre-exercise biopsy collected before the first trial was used as the sedentary reference sample for both post-exercise conditions.

Sequence effects were not explicitly included in the differential expression model. Instead, potential order effects were minimized by the randomized and balanced crossover design and by using a washout period of at least six days between trials. We have added this point as a limitation in the Discussion, noting that residual sequence, carryover, or time-related variation cannot be fully excluded. The differential expression model included horse identity to account for the repeated-measures nature of the design, and the direct comparison between positive and negative pacing was performed within this crossover framework.

7. Line 127 - 141: I found the statistics section somewhat difficult to follow, and some details that may be important for data interpretation were either unclear or missing. Furthermore, a number of analyses presented in the results section were not explained in the statistics section, e.g., the principal component analysis, the Venn diagram analysis and some of the pathway-level analyses. I acknowledge that I am not a biostatistician and may have misunderstood some aspects of the methodology employed, however it is likely that this paper will be of particular interest to exercise and muscle physiologists and I believe it is important that the statistical analysis is clearly and completely explained. Specific points to consider include:

Our response:
We thank the reviewer for this helpful comment. We agree that the statistical and bioinformatic analyses should be described more clearly and completely. We have revised the Bioinformatic and statistical analyses section to provide additional details on the analyses presented in the Results, including principal component analysis, hierarchical clustering, Venn diagram analysis, differential expression analysis, and pathway-level analyses. We have also clarified the normalization procedure, pairwise differential expression analysis, thresholds used to define differentially expressed genes, and the software/packages used for each analysis. We believe these revisions

© 2026. Published by The Company of Biologists under the terms of the Creative Commons Attribution License (https://creativecommons.org/licenses/by/4.0/).

make the analytical workflow easier to follow for readers in exercise physiology and skeletal muscle biology.

- Please explain how the repeated-measures crossover design was incorporated into the analysis and particularly how the baseline samples were handled. Providing the exact design formula used for differential expression analysis would be very useful.

Our response:
We agree that the way in which the repeated-measures crossover design and baseline samples were handled should be described more explicitly. We have revised the Bioinformatic and statistical analyses section to provide the exact design used for differential expression analysis. For differential expression analysis, each horse was treated as a repeated-measures factor, and gene expression was modeled using a design that included horse identity and experimental condition. Specifically, the DESeq2 design formula was: design = horse + condition, where condition included the pre-exercise baseline, positive-pacing, and negative-pacing samples. The baseline sample collected prior to the first exercise trial was used as the pre-exercise control condition for each horse and was compared with both post-exercise conditions. This model therefore accounted for inter-individual differences among horses while testing for the effect of exercise condition. Pairwise contrasts were then performed to compare positive pacing versus baseline, negative pacing versus baseline, and positive pacing versus negative pacing. We have also clarified in the revised manuscript that sequence effects were not explicitly included in the differential expression model. Although the order of pacing conditions was randomized and balanced, and the trials were separated by a washout period of at least 6 days, we acknowledge that residual order or carryover effects cannot be fully excluded, particularly because a single baseline biopsy was used for both exercise conditions. This point has been added to the Discussion as a limitation.

- Please clarify how repeated measurements from the same horse were accounted for within the DESeq2 design formula? This may have important implications considering the likely strong correlation between the repeated samples obtained from each horse.

Our response:
In this model, horse identity was included as a blocking factor to account for repeated measurements obtained from the same horse, whereas condition represented the experimental condition. This approach allowed us to test for the effect of condition while accounting for inter-individual differences among horses.

- At multiple times throughout the paper different "groups" were referred to, but I suggest that you instead refer to different conditions, considering that all analyses were conducted on repeated measures from the same group.

Our response:
We agree that the term “conditions” more accurately reflects the repeated-measures crossover design, as the same horses were assessed under the pre-exercise baseline, positive-pacing, and negative-pacing conditions. We have therefore revised the manuscript throughout to replace “groups” with “conditions” where appropriate.

- Line 132: How were the top 1,000 more variable genes selected for the hierarchical clustering analysis?

Our response:
We have revised the Bioinformatic and statistical analyses section to clarify how and why the top 1,000 most variable genes were selected for hierarchical clustering analysis. Genes were ranked according to the variance of their normalized expression values across all samples, and the 1,000 genes with the highest variance were selected. This number was chosen as an exploratory visualization approach to capture the major sources of transcriptomic variation while reducing the influence of low-variance genes that contribute limited information to clustering and may add noise to the heatmap. Gene expression values were then centered and scaled by gene before visualization.

© 2026. Published by The Company of Biologists under the terms of the Creative Commons Attribution License (https://creativecommons.org/licenses/by/4.0/).

- How was multiplicity handled throughout the analysis? Line 140 states that differential expression analysis was performed using DESeq2 with an FDR of < 0.05 and a minimum fold change of 1.5, however directly afterwards the manuscript states that statistical significance was defined as p < 0.05. Please clarify whether reported p-values are adjusted or unadjusted and exactly what significance criteria were applied to the different analyses.

Our response:
We agree that the description of statistical significance and multiplicity correction was unclear. We have revised the Bioinformatic and statistical analyses section to clarify the significance criteria applied to each analysis. For differential expression analysis using DESeq2, multiplicity was controlled using the Benjamini–Hochberg false discovery rate (FDR) procedure, and genes were considered differentially expressed when they met both criteria of FDR < 0.05 and a minimum fold change of 1.5. Thus, the reported significance for differential expression analysis is based on adjusted P values, not unadjusted P values. For pathway analyses, we have clarified the specific multiple-testing criteria used for each analysis. In particular, Gene Ontology over-representation analysis and gene set enrichment analysis were interpreted using FDR-adjusted P values, with significance defined as FDR < 0.05. We have also removed the general statement that "statistical significance was defined as P < 0.05" to avoid confusion, because this wording could incorrectly imply the use of unadjusted P values for RNA-seq and pathway-level analyses.

- Further to the above point, please clarify exactly how differences and similarities were identified, which will include clarification on the control condition and how it was incorporated within the model. For example, lines 172 - 176 report large number of genes differentially expressed in each exercise condition relative to control, but only 9 genes differentially expressed between pacing conditions. I understand that different analysis approaches can lead to different outcomes, but a clearer understanding of the criteria by which differences and similarities were assessed would be helpful to guide interpretation of these findings.

Our response:
We have also clarified how similarities and differences between the two exercise conditions were assessed. Similarities were assessed by identifying genes that were significantly upregulated or downregulated relative to baseline in both the positive- and negative-pacing conditions; these shared exercise-responsive genes were visualized using Venn diagrams. In contrast, pacing-dependent differences were assessed using the direct positive- versus negative-pacing contrast. Thus, the large number of genes differentially expressed in each exercise condition relative to baseline reflects the robust common response to high-intensity exercise, whereas the small number of genes differentially expressed between positive and negative pacing indicates that the pacing-dependent transcriptional effect was modest.

- The interpretation of "condition-specific" genes based on the Venn diagram analysis was also somewhat unclear. From what I understand, this analysis represents genes that were differentially expressed in one exercise-versus-control comparison, but this does not necessarily imply a statistically significant difference between pacing conditions.

Our response:
We agree that genes shown as unique to one exercise condition in the Venn diagram analysis do not necessarily indicate a statistically significant difference between the positive- and negative-pacing conditions. Rather, these genes represent genes that were detected as differentially expressed relative to the sedentary condition in only one pacing condition. To avoid overinterpretation, we have revised the manuscript to remove or clarify the term "condition-specific" in relation to the Venn diagram analysis. We now refer to these genes as "genes differentially expressed relative to the sedentary condition in only one pacing condition." We have also clarified that statistically significant pacing-dependent differences were defined only by the direct comparison between positive and negative pacing, which identified nine differentially expressed genes. This revision provides a more accurate interpretation of the Venn diagram analysis and avoids implying that genes appearing in only one part of the Venn diagram were significantly different between pacing conditions.

8) The current discussion largely focuses on the observed differences between the pacing strategies. While the broad conclusion of the a largely conserved transcriptional response, with

© 2026. Published by The Company of Biologists under the terms of the Creative Commons Attribution License (https://creativecommons.org/licenses/by/4.0/).

some potential modulated expression of limited genes seems reasonable based on the reported data, I do suggest caution throughout in interpreting individual gene differences, considering the large potential for biological variation in studies of this kind. Greater clarity on the statistical approach employed, and particularly the points described above will be very useful in better understanding the discussion. It is possible that some points may require some reconsideration depending on the authors response to the above points.

Our response:
We agree that the small number of pacing-dependent gene-level differences should be interpreted cautiously, particularly given the potential for biological variation in this type of repeated-measures study. We have therefore revised the Discussion to emphasize the largely conserved transcriptomic response between pacing strategies and to reduce overinterpretation of individual gene differences. Although we retained concise literature-based context for genes such as *RP1*, *POSTN*, *MAP1A*, and *ANGPTL1*, we have shortened and tempered these sections and now state that the functional significance of these limited differences remains uncertain and requires further validation. We have also expanded the Methods section to describe the statistical approach more clearly, and clarified the study design including the limitations. These revisions provide a more cautious and balanced interpretation of the limited pacing-dependent differences observed in the present study.

Second decision letter

MS ID#: bio.062724R1

MS Title: Conserved skeletal muscle transcriptomic responses to pacing strategies in Thoroughbred horses

Authors: Kenya Takahashi, Kazutaka Mukai, Takanaga Shirai, Yusaku Ebisuda, Fumi Sugiyama, Toshinobu Yoshida, Hideo Hatta and Yu Kitaoka

I am happy to tell you that your manuscript has been accepted for publication in Biology Open, pending our standard publication integrity checks. It was accepted on 26th June 2026.

© 2026. Published by The Company of Biologists under the terms of the Creative Commons Attribution License (https://creativecommons.org/licenses/by/4.0/).
